# Editorial: Tumor-Derived Extracellular Vesicles: Protocols, Models, and Clinical Evidence

**DOI:** 10.3389/fonc.2016.00230

**Published:** 2016-10-25

**Authors:** Renaud Seigneuric, Carmen Garrido

**Affiliations:** ^1^LNC UMR866, Univ. Bourgogne Franche-Comté, Dijon, France; ^2^LNC UMR866, INSERM, Equipe Labellisée “Ligue Nationale Contre le Cancer”, Dijon, France; ^3^Centre Georges François Leclerc, Dijon, France

**Keywords:** extracellular vesicles, translational medical research, cancer, exosome, biomarkers, targeted therapy, liquid biopsy, nanoparticles

**The Editorial on the Research Topic**

**Tumor-Derived Extracellular Vesicles: Protocols, Models, and Clinical Evidence**

The current frontiers in Oncology are at the nanoscale (1). Within this nanoworld, new instruments and their related protocols enable the discovery or the improved characterization of bio-objects including extracellular vesicles (EVs). EVs, secreted by cells into the extracellular environment, include exosomes, microvesicles, and apoptotic bodies. Thus, EVs range from approximately 40 nm to a few millimeters in size (2). Deriving from the endosomal compartment, exosomes are cell-secreted nanovesicles within the 70–150 nm diameter range that have recently aroused a great interest in the scientific and clinical community for their roles in intercellular communication in almost all physiological and pathological processes (2).

The growing interest for exosomes in biology stems from the fact that these vesicles: (i) are ubiquitous (e.g., from plants to eukaryotic cells); (ii) contain a variety of molecules including signal peptides, mRNAs, microRNAs (miRNAs), proteins, and lipids; (iii) are involved in local and systemic cell communication (exosomes can bind to cells through receptor–ligand interactions, can also pass through the blood–brain barrier and the placenta); (iv) all living cells can release exosomes including tumor cells; and (v) can be detected in body fluids (e.g., blood and urine).

In Oncology, the many characteristics and roles of circulating tumor-derived exosomes are being elucidated (3–7). This research topic aims at providing recent findings and reviews to contribute to the maturation of this emerging field with high clinical potential.

Exosomes carry biologically active molecules including non-coding RNAs (ncRNAs) that are usually divided into two major groups according to their length: small ncRNAs (below 200 nt) defined as miRNAs and long ncRNAs (lncRNAs; above 200 nt). miRNAs mostly act on RNA by silencing or posttranscriptionally regulating gene expression, whereas lncRNAs participate in imprinting and gene dosage regulation, using diverse molecular mechanisms. In their review, Lopatina et al. mainly focus on the role of EV-transferred RNAs carried by tumor-derived and mesenchymal stem cells (MSC)-derived EVs and how they can alter tumor microenvironment. They gather evidences suggesting that ncRNAs can not only modulate gene expression locally but also systemically. They also show that depending on their biological properties and content, EVs are involved in cancer initiation, progression, and premetastatic niche formation. It is becoming evident that EVs may transfer not only functional ncRNA but also DNA, thus modifying gene expression in recipient cells and further extending EVs communication modalities. In line with the article of Al-Nedawi, they pinpoint the dual role of exosomes and stress out the probable importance of the environment.

The group of Takahashi et al. describes methods for the isolation of EVs from the cell culture supernatant and from human serum and the extraction of extracellular RNA by digital PCR (dPCR). Quantitative real-time polymerase chain reaction (qPCR) is a widely adopted technique since its invention in the mid 80s (8, 9). dPCR, developed in the late 90s, extends these applications while providing an increased sensitivity (10). Each sample is partitioned into thousands (~10^4^ to ~10^6^) nanoliter to picoliter-sized droplets moving through microfluidics chips where a PCR either occurs (1) or not (0). dPCR also provides interesting improvements including increased precision and reproducibility as well as absolute quantification (thus avoiding the need of reference genes). The authors provide an interesting example of dPCR quantitation of low abundance transcripts that can be present within EVs.

In his opinion article, Al-Nedawi discusses the Janus-faced implications of exosomes in cancer biology. Indeed, some controversies arise from the fact that exosomes can transfer tumor-promoting molecules (e.g., oncoproteins) and tumor suppressors. The author intends to make sense of seemingly opposite findings such as tumor suppressor PTEN or VEGF receptor-2 (VEGFR2) triggering the angiogenic switch to initiate angiogenesis. Exosomes can carry mRNAs and miRNA (miR) that can have various effects on tumor progression by modulating tumor microenvironment. An interesting cross talk implying miR-122 is discussed. Also, the double-edge sword implications relating tumor-associated macrophages, VEGF, miR-150, miR-142, and miR-223 are detailed.

In their research article, Gong et al. tackle the issue of drug resistance and metastasis. Generally, these two issues are addressed separately. However, recent evidence associates resistance with an enhanced metastatic capacity. The authors, who previously described the intercellular transfer of drug resistance *via* submicron vesicles called microparticles, now propose that these vesicles derived from drug-resistant cells are also involved in the intercellular transfer of components to enhance the migration and invasion capacity of cells. As such, they might be a channel between resistance and metastasis. Using microarrays, they identify regulatory miRNAs. Among them, miR-503 is inversely associated with metastasis, as demonstrated using wound healing/scratch migration assays and matrigel-coated transwell invasion assays. Their functional characterization goes beyond the direct transfer of vesicle components.

The review of Zocco et al. discusses recent reports about the clinical utility and current limitations of exosomes and microvesicles for *thera*peutic and diag*nostic* applications (theranostics). The authors provide a summary of recent preclinical and clinical studies on EV-shuttled biomarkers that may prove useful for screening and/or early diagnosis. They also discuss current technological challenges that the EV community is facing for the development of EV-based diagnosis approaches. The central issue of EV isolation protocols is detailed. Being sub-optimal, our current protocols produce EVs of variable yield, purity (origin), and integrity, making them poorly compatible with routine use for diagnostic purposes. As therapy is concerned, EVs may be exploited as biomimetic nanocarriers. Finally, the authors debate the two main limitations of these studies: the lack of standardized protocols for isolation of clinical grade EVs (sub)populations and the partial understanding of the mechanisms involving EVs.

This research topic collates research findings that illustrate tightly regulated EVs feedback loops necessary within a physiological context (dynamical equilibrium) and whose deregulation can lead to pathological disorders. The importance of cells’ environment is highlighted. As nanoshuttles of biomarkers and/or anti-tumor drugs, exosomes open new avenues for the clinical management of cancer. However, the lack of standardized isolation protocols in this emergent field currently hampers clinical studies.

## Author Contributions

All authors listed have made substantial, direct, and intellectual contribution to the work and approved it for publication.

## Conflict of Interest Statement

The authors declare that the research was conducted in the absence of any commercial or financial relationships that could be construed as a potential conflict of interest.
